# AKR1C3–PKM2–oxidative phosphorylation axis drives prostate cancer radioresistance via UBE2T upregulation

**DOI:** 10.1038/s41419-026-08666-5

**Published:** 2026-03-30

**Authors:** Jinyu Zhang, Jiongzheng Li, Yufei Yan, Binghuan Wang, Ruojia Wang, Xiaoli Cui, Yang Zhan, Zuowen Liang, Jing Li

**Affiliations:** 1 Department of Pharmacology, College of Basic Medical Sciences, Jilin University, Changchun, PR China; 2 Department of Pharmacology, College of Pharmacy, Beihua University, Jilin, PR China; 3 Department of Biochemistry and Molecular Biology, College of Life Sciences, Jilin University, Changchun, PR China

**Keywords:** Prostate cancer, Cancer metabolism

## Abstract

Radioresistance is one of the primary causes of prostate cancer treatment failure and post-radiotherapy progression. However, there is currently a lack of effective targets to increase radiotherapy sensitivity and inhibit malignant progression. We identified AKR1C3 as a potential key target associated with radioresistance and malignant progression through integrated bioinformatic analysis of RNA sequencing (RNA-seq) data from prostate cancer clinical samples in The Cancer Genome Atlas (TCGA) and Gene Expression Omnibus (GEO) databases. The promotion of radioresistance by AKR1C3 in both AR-positive and AR-negative prostate cancer cells was further validated through in vivo and in vitro experiments. Mechanistic studies revealed that AKR1C3 can bind to PKM2 and accelerate its degradation, thereby inhibiting glycolytic flux and enhancing oxidative phosphorylation (OXPHOS). Increased OXPHOS boosts ROS production, which further promotes NRF2 nuclear translocation, activating the transcription of DNA repair protein UBE2T. This enhanced DNA damage repair ability enables prostate cancer cells with high AKR1C3 expression to exhibit greater resistance to radiotherapy. In summary, this study reveals the molecular mechanism by which AKR1C3 is involved in metabolic reprogramming to promote radioresistance in prostate cancer through PKM2/UBE2T. These findings indicate that targeting AKR1C3 has potential for overcoming radioresistance, providing novel insight into the clinical treatment of prostate cancer.

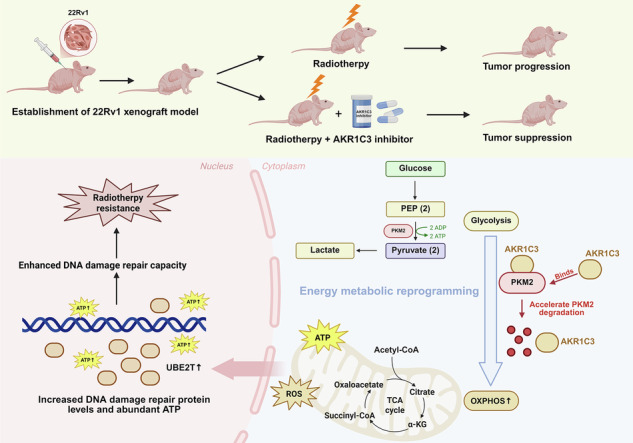

## Introduction

Prostate cancer (PCa) is a prevalent malignancy in men and its development is tightly linked to androgens [1]. According to cancer statistics from 2025, prostate cancer accounts for approximately 30% of all newly diagnosed cancer cases among men in the United States, representing the most commonly diagnosed malignancy in men. Additionally, prostate cancer contributes to 11% of cancer-related deaths, ranking as the second leading cause of cancer mortality among men [2]. Clinically, prostate cancer is classified into stages I to IV, each with well-established standard therapeutic protocols. Radiotherapy (RT) is an effective treatment option for prostate cancer at all stages, particularly in patients with localised disease [3]. However, some patients develop radioresistance following standalone or combined radiotherapy [4]. If early RT and/or androgen deprivation therapy (ADT) fails, the disease may progress to castration-resistant prostate cancer (CRPC), which is incurable [5]. The mechanisms driving radioresistance in PCa remain incompletely defined and effective clinical targets to prevent or mitigate radioresistance and delay tumour progression are lacking. Thus, elucidating the biological basis of radioresistance and devising innovative therapeutic strategies holds profound clinical implications for disease management.

Radiotherapy induces DNA damage in tumour cells by ionising radiation (IR), triggering the DNA damage response (DDR) to repair damage and sustain cell survival [6]. In radioresistant cells, DDR proteins such as PRKDC, RAD51 and UBE2T are frequently overexpressed, thereby increasing the ability of cells to repair DNA [7–9]. Given that therapeutic radiation causes damage within minutes, de novo protein synthesis cannot account for this protective response. We hypothesize that intrinsic factors increase DDR protein expression in cancer cells before radiotherapy, which enhances their ability to repair DNA and confers intrinsic resistance to damage caused by exogenous factors. On the basis of these findings, our study sought to pinpoint genes or proteins closely related to PCa radiosensitivity and progression. We conducted an integrated bioinformatic analysis of public datasets and identified aldo-keto reductase 1C3 (AKR1C3) overexpression as significantly correlated with both radioresistance and disease progression in PCa.

AKR1C3 plays a well-established role in PCa malignant progression through dual mechanisms. Enzymatically, AKR1C3 leverages NADH and NADPH as coenzymes to metabolise steroids and synthesise endogenous androgens, thereby activating the androgen receptor (AR) signalling pathway, which transcriptionally upregulates multiple DNA damage response genes and promotes DNA repair in prostate cancer [10, 11]. Nonenzymatically, AKR1C3 was reported to bind AR-V7 and Siah2, stabilising them against ubiquitin-mediated degradation and thereby promoting PCa progression [12, 13]. Notably, AKR1C3-mediated radioresistance is also observed in AR-negative malignancies, including oesophageal carcinoma and non-small cell lung cancer [14, 15]. These findings indicate that, in addition to promoting tumour progression through AR activation, AKR1C3 may drive AR-independent radioresistance mechanisms.

Emerging evidence indicates a close relationship between energy metabolic reprogramming and radioresistance. Radiotherapy may induce metabolic reprogramming in tumour cells and this metabolic reprogramming may also contribute to the development of radioresistance [16, 17]. While the Warburg effect highlights the preference of cancer cells for aerobic glycolysis, accumulating data indicate that the ability of cancer cells, especially cancer stem cells, to perform oxidative phosphorylation (OXPHOS) remains [18]. As a core bioenergetic pathway, OXPHOS has strong mechanistic links to DNA repair proficiency and radiosensitivity [19–21]. Acute and chronic DNA damage promote mitochondrial fatty acid oxidation (FAO) and OXPHOS and Fanconi anaemia (FA) stem cells depend on OXPHOS to cope with oxidative DNA damage under stress [22]. Furthermore, studies have demonstrated that the inhibition of mitochondrial OXPHOS can increase radiosensitivity. IR-TAM, a conjugate of the heptamethine cyanine dye IR-68 and tamoxifen (TAM), selectively suppresses OXPHOS activity, which can not only potentiate radiotherapy efficacy but also attenuate radiation-induced pulmonary fibrosis [23].

However, despite the established role of AKR1C3 as a key enzymatic regulator of prostatic steroid metabolism, its potential involvement in prostate cancer energy metabolism remains unexplored. Consequently, investigating this topic may offer novel mechanistic insights for counteracting radioresistance and malignant progression in prostate cancer. In this study, we identified AKR1C3 as a dual target for enhancing prostate cancer radiosensitivity and preventing malignant progression. We discovered that AKR1C3 regulates OXPHOS in prostate cancer by binding to PKM2 and accelerating PKM2 degradation, thereby promoting the upregulation of DDR proteins such as UBE2T. This metabolic reprogramming confers intrinsic resistance to exogenous genotoxic stress in AKR1C3-high prostate cancer cells, fundamentally underpinning their radioresistance. Through in vivo experiments, we assessed the feasibility of using AKR1C3 inhibitors to address radioresistance in prostate cancer, providing novel approaches for clinically managing radioresistance and malignant progression and advancing personalised treatment strategies for prostate cancer.

## Methods

### Identification of genes associated with radioresistance and malignant progression in prostate cancer

Differentially expressed gene (DEG) analysis and survival analysis were performed using data from the TCGA database. For disease-free survival (DFS) and pathological stage analyses, clinical and gene expression data from 498 prostate cancer patients were used to evaluate the associations between specific gene expression levels and DFS or pathological T (pT) stage. For the analysis of DEGs related to radiotherapy sensitivity, a total of 41 patients with localised prostate cancer who underwent radiotherapy and had documented treatment responses were included. Patients were divided into two groups: those with a ‘complete response’ were classified as radiotherapy sensitive and the remainder were classified as radiotherapy nonsensitive. Additionally, the GSE200879 dataset from the GEO database was used to identify DEGs between CRPC (*n* = 13) and typical prostate cancer patients (*n* = 115) [24]. Genes with a fold change (FC) ≥ 1.75 and *p* < 0.05 were considered differentially expressed. The common DEGs in the datasets were further analysed for their associations with DFS and pT stage. Genes with high expression associated with shortened DFS and more advanced pT stage were identified as candidate targets involved in radioresistance and malignant progression. All differential gene expression analyses were performed using the limma package in R [25].

### Cell culture, ionising radiation and animal experiments

The human prostate cancer cell lines LNCaP, 22Rv1 and PC-3 were obtained from the Chinese Academy of Sciences Cell Bank (Shanghai, China) and were used at passages fewer than 10. LNCaP is an androgen-dependent cell line that expresses a functional AR and exhibits low AKR1C3 expression, indicating a low-to-moderate malignancy phenotype. In contrast, 22Rv1 is an androgen-independent cell line with high AR and AKR1C3 expression and displays a more aggressive phenotype. PC-3 is an androgen-independent prostate cancer cell line that lacks functional AR, resulting in a phenotype that is unresponsive to androgenic stimulation. LNCaP, 22Rv1 and PC-3 cells were routinely cultured in RPMI-1640 medium (Gibco) or DMEM medium (Gibco), each supplemented with 10% foetal bovine serum (FBS; HyClone) and 1% penicillin‒streptomycin (Biosharp). Cultures were maintained at 37 °C in a humidified incubator with 5% CO₂. All experiments were conducted using cells in the logarithmic growth phase.

Irradiation (IR) was administered using a SIEMENS therapeutic X-ray machine (RS 2000 PLUS). The dose rate for cell irradiation was 1.00 Gy/min, whereas the dose rate for animal irradiation was 2.22 Gy/min.

Male BALB/c-nu nude mice (6-8 weeks old, weighing 20 ± 2 g) were acclimated for 1 week prior to the experiments. To establish the 22Rv1 and PC-3 subcutaneous xenograft models, 5 × 10^6^ 22Rv1 or PC-3 cells were suspended in 50 µL of phosphate-buffered saline (PBS) and 50 µL of phenol red-free Matrigel and subcutaneously injected into the right shoulder of each mouse. When the tumor volume reached approximately 200 mm³ (21 days post-inoculation), the tumor-bearing mice were randomly assigned to different groups. The 22Rv1 xenograft model was divided into: control group (Con), radiation therapy group (RT), ASP9521 monotherapy group (ASP9521) and radiation therapy combined with ASP9521 group (RT + ASP9521); another set of 22Rv1 models was divided into: control group (Con), radiation therapy group (RT) and radiation therapy combined with siAKR1C3 group (RT+siAKR1C3). The PC-3 xenograft model was divided into: control group (Con), radiation therapy group (RT) and radiation therapy combined with AKR1C3 group (RT + AKR1C3). The minimum number of mice per group was 6. From the first day after grouping, the ASP9521 and RT + ASP9521 groups received daily gavage of 3 mg/kg ASP9521, while the control and RT groups received the same dose of CMC-Na for 21 days; the RT+siAKR1C3 and RT + AKR1C3 groups were intratumorally injected with 80 µL of the corresponding transfection mixture every 3 days, while the control and RT groups received the same dose of transfection reagent, for a duration of 21 days.

The radiotherapy and combination groups were irradiated twice, on days three and nine post-grouping, with a total dose of 20 Gy (BED = 40 Gy) at a rate of 2.22 Gy/min. The mice were anesthetized with 1% sodium pentobarbital (0.1 mL/20 g) and securely fixed during irradiation. Lead shielding was applied to ensure that only the tumor region was exposed to radiation, protecting the surrounding tissues.

On day 21, the mice were euthanized via isoflurane anaesthesia followed by CO₂ inhalation. Subcutaneous tumours were excised and weighed and their volumes were calculated using the following formula: volume = 0.52 × width² × length. Tumours were then processed for Western blot (WB) and TUNEL analyses.

All animal experiments conducted in this study were approved by the Ethics Committee of the College of Pharmacy, Beihua University (Approval No. 2024082301). All animal experiments were performed in accordance with relevant guidelines and regulations.

### Gene overexpression and knockdown

Transfections were performed using the Starvio Plasmid DNA Transfection Reagent for plasmid or the Starvio siRNA Transfection Reagent for small interfering RNA (siRNA) (both from STARVIO, Shanghai, China). For in vivo transfections, the Entranster™-in vivo reagent (Engreen, Beijing, China) was used. The control groups were transfected with empty vector plasmids or negative control siRNA. All the transfection procedures and reagent quantities adhered to the manufacturer’s protocols. The siRNA sequences used in this study are listed in Table S1.

### Western blot analysis

Whole-cell lysates were subjected to SDS–PAGE, transferred to PVDF membranes and blocked in 5% nonfat milk (TBST) for 1 h at room temperature. The membranes were incubated overnight at 4 °C with the following primary antibodies: anti-γ-H2AX rabbit polyclonal antibody (pAb) (YP-Ab-01248; UpingBio), anti-UBE2T rabbit monoclonal antibody (mAb) (ab179802; Abcam), anti-PKM2 rabbit mAb (F0295; Selleck), anti-VCAN mouse mAb (YP-mAb-12480; UpingBio), anti-COL1A1 mouse mAb (YP-mAb-12480; UpingBio), anti-CRIP2 mouse mAb (YP-mAb-18822; UpingBio), anti-ETV1 mouse mAb (YP-mAb-01698; UpingBio), anti-AMIGO2 mouse mAb (YP-mAb-07231; UpingBio), anti-CHGB mouse mAb (YP-mAb-07705; UpingBio), anti-AKR1C3 mouse mAb (A6229; Sigma‒Aldrich) and anti-β-actin mouse mAb (T0022; Affinity). The membranes were subsequently incubated with secondary antibodies (ProteinTech, Wuhan, China) at a 1:5000 dilution for 2 h at room temperature, followed by chemiluminescence detection.

### Proteomics

LNCaP-AKR1C3 cells and their parental LNCaP counterparts were cultured for 48 h under standard conditions, harvested, lysed for protein extraction and stored at −80 °C until proteomic analysis. Quantitative 4D label-free proteomic profiling was carried out by Jingjie PTM-Biolab (Hangzhou, China) following the company’s validated workflow.

### Immunofluorescence (IF)

A total of 1 × 10⁴ 22Rv1 cells were seeded in 24-well plates. The cells were fixed with 4% paraformaldehyde for 30 min, permeabilized with 0.2% Triton X-100 and blocked with 5% donkey serum in phosphate-buffered saline (PBS) to minimise nonspecific binding. The primary antibodies anti-PKM2 rabbit mAb (1:100, F0295; Selleck) and anti-AKR1C3 (1:50, A6229; Sigma‒Aldrich) were applied overnight at 4 °C. PKM2 and AKR1C3 expression were visualised using Alexa-Fluor 594-conjugated and Alexa-Fluor 488-conjugated secondary antibodies and nuclei were stained with DAPI. Images were acquired using a laser scanning confocal microscope (FV3000, Olympus).

### Colony formation assay

Cells were subjected to 2 Gy IR at a dose rate of 1 Gy/min. Following IR, 1600 cells per well were plated in 6-well plates and incubated for 14 days, with fresh medium replaced every 3 days. After incubation, the cells were fixed with 4% paraformaldehyde for 15 min and stained with 0.1% crystal violet for 30 min. The plates were gently rinsed with deionized water to remove excess dye and allowed to dry. Colonies were counted under a microscope and the colony formation rate was calculated as (number of colonies/number of seeded cells) × 100%. Differences between the irradiated and control groups were analysed to evaluate the impact of radiation on cell proliferation.

### Comet assay

Cells were exposed to 5 Gy IR at a dose rate of 1 Gy/min and incubated for 4 h. Following incubation, the cells were harvested and subjected to the comet assay. Cells were embedded in low-melting-point agarose, lysed,= and subjected to DNA unwinding and horizontal electrophoresis. After electrophoresis, the DNA was stained with a fluorescent dye and observed under a fluorescence microscope (IX71, Olympus). The olive tail moment was calculated for each group to assess DNA damage using the following formula:

Tail DNA (%) = 100 × (Tail DNA Intensity/Total Cell DNA Intensity)

Olive tail moment = tail DNA (%) × tail moment length

### ATP content measurement

The cells were lysed and the supernatant was collected after centrifugation. The ATP detection working solution (Beyotime Biotechnology, China) was added to a 96-well plate, followed by the addition of the cell lysate. After gentle mixing and incubation at 37 °C for 5 min, luminescence was measured using a microplate reader. The ATP content was calculated on the basis of a standard curve and normalised to the total protein concentration.

### Measurement of reactive oxygen species (ROS) levels

The intracellular ROS levels were evaluated using a dihydroethidium (DHE) fluorescent probe (Beyotime, China). The cells were seeded in culture dishes and, after reaching the appropriate confluency, incubated with DHE at 37 °C for 30 min in the dark. After incubation, the cells were washed with prewarmed PBS to remove excess probe. Fluorescence images were then acquired using a fluorescence microscope (IX71, Olympus).

### Gene ontology (GO) and Kyoto Encyclopedia of Genes and Genomes (KEGG) enrichment analyses

GO [26] and KEGG [27] pathway enrichment analyses were performed to functionally annotate and explore the biological pathways associated with the upregulated DEGs identified by proteomics. The analyses were performed using the ‘clusterProfiler’ package in R [28], on the basis of official gene annotation data. The GO enrichment analysis included the biological process (BP), cellular component (CC) and molecular function (MF) categories. KEGG pathway analysis was used to identify enriched signalling pathways.

### Spearman correlation analysis

RNA-seq data and clinical metadata from 497 prostate cancer patients were obtained from the TCGA. Samples lacking either expression profiles or clinical annotations were excluded. Pathway activity scores were derived through single-sample gene set enrichment analysis (ssGSEA) implemented in the R GSVA package (method = ‘ssgsea’) using predefined pathway gene sets [29]. Finally, Spearman’s rank correlation was used to assess associations between individual gene expression levels and pathway enrichment scores. Genes with a correlation coefficient greater than 0.5 and a *p*-value less than 0.05 were deemed to have significant pathway associations.

### Reverse transcription–quantitative polymerase chain reaction (RT–qPCR)

Total RNA was extracted with a TIANGEN RNA extraction kit (TIANGEN Biotech, Beijing, China). RNA was reverse-transcribed using a TransGen Biotech reverse transcription kit (TransGen, Beijing, China) according to the manufacturer’s instructions. qPCR was performed with a 2X SYBR Green master mix (Roche, Basel, Switzerland) on a real-time PCR system under standard cycling (95 °C 30 s; 40 cycles of 95 °C 5 s, 60 °C 30 s), followed by melt-curve analysis. Relative expression was calculated by the 2^-ΔΔCt^ method using GAPDH as the reference gene; primer sequences are provided in Table S1.

### Coimmunoprecipitation (CoIP) and mass spectrometry analysis

Cells were lysed in ice-cold IP buffer (Beyotime, China) with protease and phosphatase inhibitors. After centrifugation, the supernatant was collected and incubated with anti-Myc magnetic beads (Biolinkedin, Shanghai, China) for 2 h at 4 °C with gentle rotation. The beads were then washed three times with lysis buffer to remove nonspecifically bound proteins. The bound proteins were eluted by boiling the beads in SDS loading buffer. For mass spectrometry analysis, the peptides were analysed by data-dependent acquisition using a Q Exactive HF-X mass spectrometer (Thermo Scientific). Liquid chromatography–tandem mass spectrometry (LC‒MS/MS) analysis and raw data processing were performed by Bioprofile (Shanghai, China). Protein identification and quantification were conducted using standard database search algorithms against the appropriate species-specific protein database.

### TUNEL staining

TUNEL staining was performed on tumour paraffin sections. Sections were deparaffinized, rehydrated and incubated with proteinase K (20 μg/mL) at 37 °C for 20 min. Following PBS washes, sections were incubated with TUNEL assay solution (50 μL) at 37 °C for 60 min in the dark. After additional PBS washes, sections were counterstained with DAPI-containing antifade mounting medium and visualised using a fluorescence microscope (IX71, Olympus).

### Basal oxygen consumption rate (OCR) measurement

Basal OCR was measured using the BBoxiProbe® R01 Oxygen Consumption Assay Kit (BestBio, China) according to the manufacturer’s instructions. The R01 probe was diluted 1:25 in prewarmed culture medium and added at 100 μL per well to a black, clear-bottom 96-well plate, followed by incubation at 37 °C for 30 min. Subsequently, 100 μL of oxygen-sealing solution was gently overlaid per well to establish a closed measurement system while avoiding bubbles. Fluorescence was recorded on a Synergy H1 multi-mode microplate reader (Agilent, USA) at 37 °C (excitation 460 nm, emission 600 nm) every 10 min for 120 min. After blank subtraction, basal OCR was calculated as the slope of fluorescence versus time within the linear range.

### Cycloheximide (CHX) chase assay

Cells were treated with cycloheximide (CHX, 50 μg/mL) to block protein synthesis. Cells were harvested at 0, 2, 4, 6 and 8 h, lysed in RIPA buffer.

### MG132 rescue experiment

Cells were pretreated with MG132 (10 μM) and CHX (50 μg/mL) to inhibit proteasomal degradation and block protein synthesis. Cells were incubated at 0, 2, 4, 6, 8 and 10 h, lysed in RIPA buffer for subsequent protein extraction. Protein levels were determined by Western blot analysis.

### Dual-luciferase reporter assay

The dual-luciferase reporter assay was used to assess the transcriptional activity of the UBE2T promoter regulated by Nrf2. Cells were seeded in 24-well plates and co-transfected with Firefly luciferase and Renilla luciferase reporter plasmids. After 48 h of transfection, cells were collected and Firefly luciferase and Renilla luciferase activities were measured. The Firefly luciferase activity was normalised to Renilla luciferase activity to account for transfection efficiency and cell viability. Results were expressed as relative luciferase units.

### Prediction of promoter transcription factor binding sites

Promoters were defined as the 2000-bp region upstream of the annotated transcription start site of each human gene (genome assembly GRCh38/hg38). Position weight matrices were obtained from the JASPAR database. Genomic sequences were scanned with the JASPAR ‘scan’ tool using default settings and a relative score threshold of 0.80; only matches with a relative score ≥0.80 were retained.

### Protein interaction site prediction method

Protein interaction sites were predicted using AlphaFold 3, a deep learning-based method for protein structure prediction. The 3D structures of target proteins were generated by AlphaFold 3 based on their amino acid sequences. To evaluate the reliability of the predicted interaction sites, we incorporated the post-translational modification (PTM) and the improved post-translational modification (iPTM) values derived from AlphaFold 3 outputs. A pTM score above 0.5 indicates that the overall predicted fold for the complex might be similar to the true structure, while the iPTM score measures the accuracy of the predicted relative positions of the subunits within the complex. Values below 0.6 suggest a likely failed prediction.

### Statistical analysis

The data are presented as the means ± standard deviations (SDs). Statistical significance between two groups was assessed using two-sided Student’s *t* tests and normality was tested using the Shapiro-Wilk test. For comparisons involving multiple groups, one-way analysis of variance (ANOVA) followed by Tukey’s post hoc test was performed. Multiple comparison corrections were applied where appropriate. The statistical analysis was performed with blinding to group allocation and Levene’s test was used to assess the homogeneity of variances between groups. A *p*-value ≤ 0.05 was considered statistically significant. The statistical values are marked as data points in the figure. Statistical analyses were conducted using GraphPad Prism 9.0.

## Results

### AKR1C3 is associated with radioresistance and malignant progression in prostate cancer

**Fig. 1:**
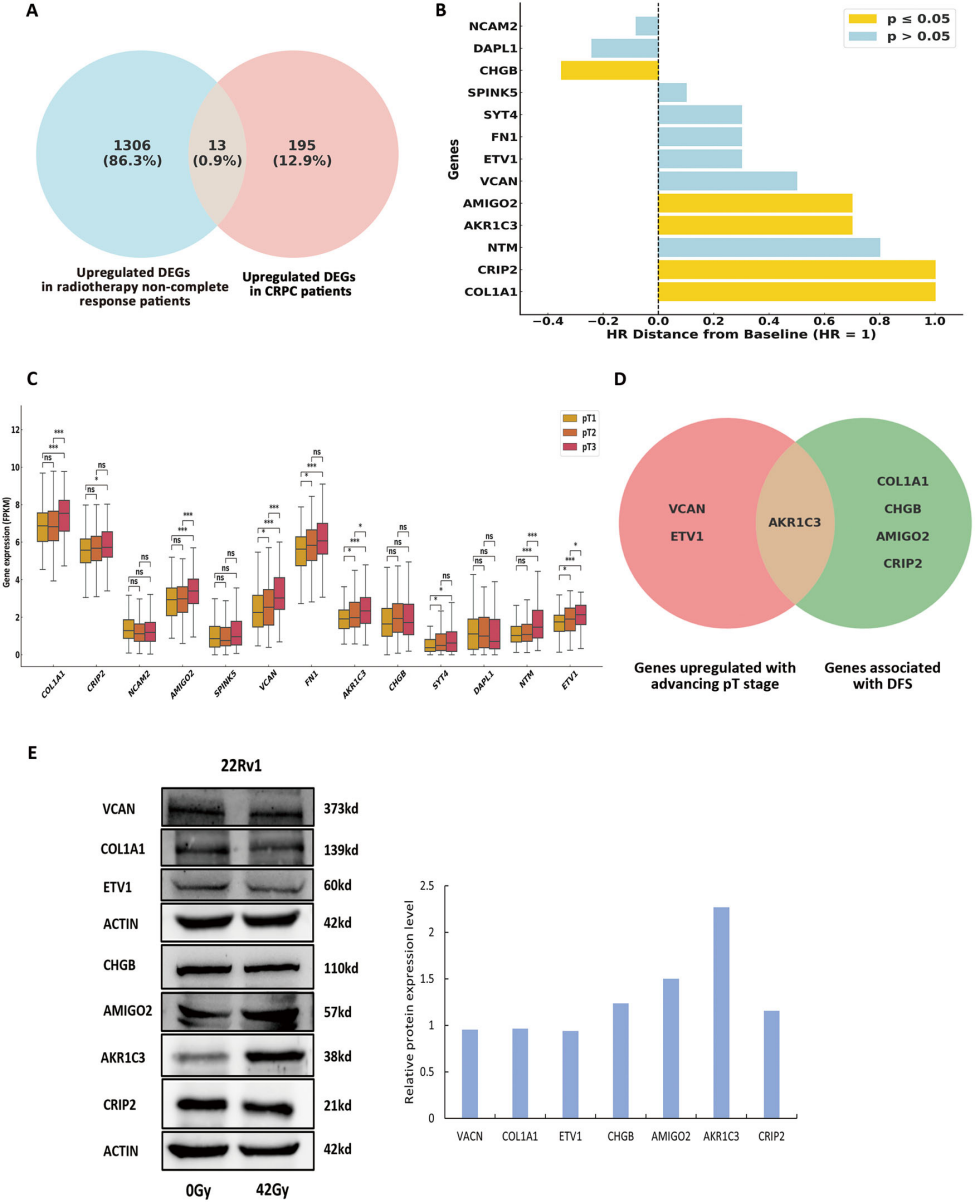
Identification of genes linked to radioresistance and malignant progression in prostate cancer. **A** Venn diagram displaying the intersection of genes upregulated in patients with poor response to radiotherapy and genes elevated in patients with CRPC. **B** Forest plot displaying hazard ratios (HRs) for DFS associated with the expression of 13 genes. The yellow bars indicate significant HRs (*p* ≤ 0.05) and the blue bars represent nonsignificant HRs (*p* > 0.05). **C** Box plot demonstrating the 13 candidate genes' expression levels across different pT stages. **D** Venn diagram showing genes whose expression is upregulated with increasing pT stage and is associated with DFS. **E** Western blot analysis of the expression levels of the seven candidate genes in surviving cells compared with parental cells. Statistical significance: *p < 0.05, **p < 0.01, ***p < 0.001, ns not significant.

To identify genes related to radioresistance and malignant progression in prostate cancer, we analysed relevant clinical sample data from the TCGA and GEO databases. This involved screening DEGs between samples from radiotherapy complete response patients and non-complete response patients, as well as DEGs between samples from localised prostate cancer patients and those with CRPC. By comparing the upregulated DEGs from both DEG analyses, we identified 13 common genes (Fig. 1A). To further characterise the clinical significance of these 13 genes, we analysed the associations between their expression patterns and DFS and across pT stages. We considered candidate genes whose high expression was associated with low DFS and whose expression increased with advancing pT stage. Our analysis identified seven genes that met at least one of these criteria, but only AKR1C3 fulfilled both criteria (Fig. 1B–D). To identify the gene most closely linked to radioresistance, we subjected 22Rv1 cells to continuous irradiation at 3 Gy per day for 14 days, with a total dose of 42 Gy and compared the protein expression levels of the seven candidate genes between the surviving cells and parental controls (Fig. 1E). Notably, AKR1C3 expression was significantly elevated in surviving cells compared with parental cells, indicating that AKR1C3 contribute to both radioresistance and malignant progression in prostate cancer.

### AKR1C3 is positively correlated with radioresistance in prostate cancer

**Fig. 2:**
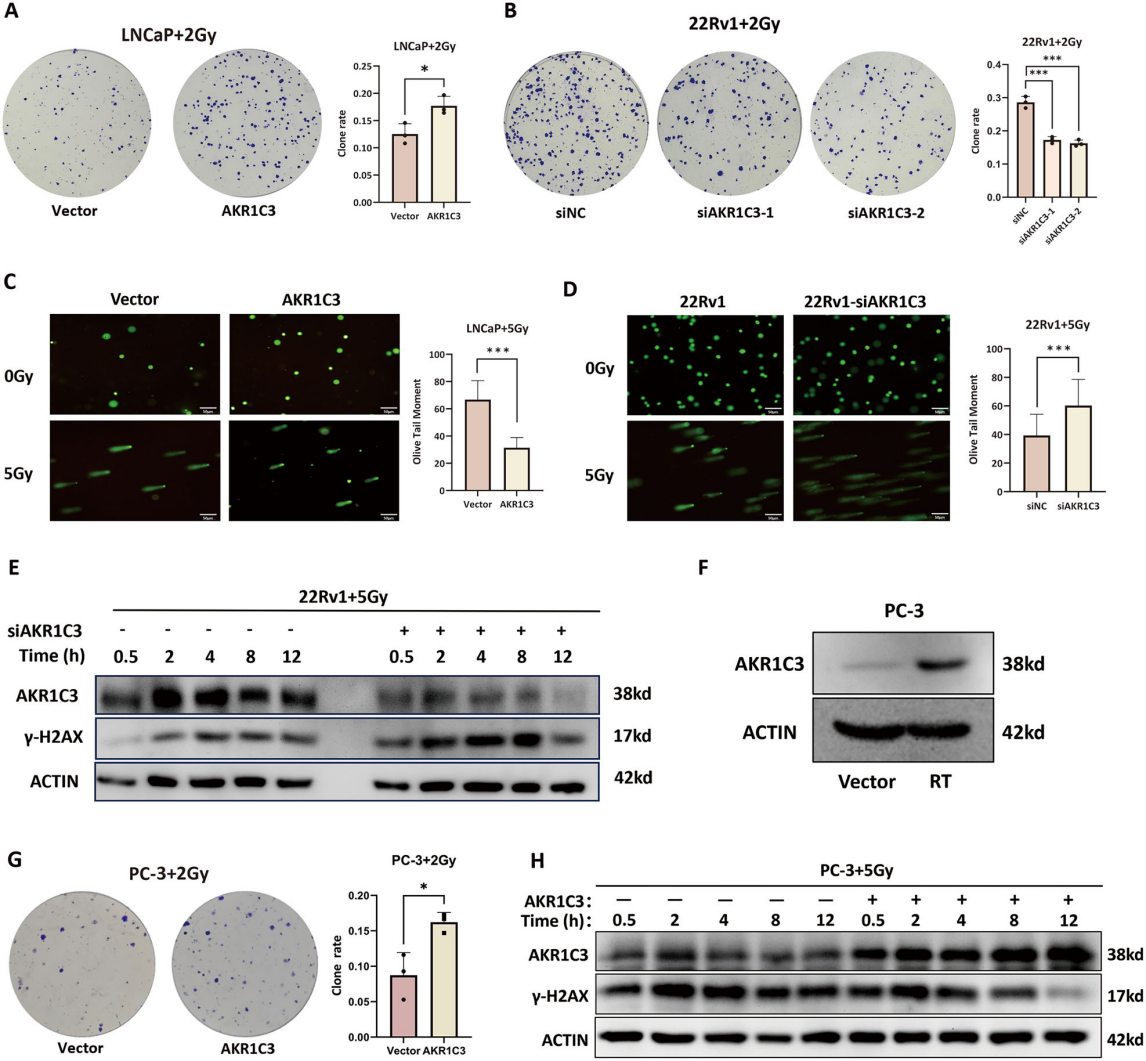
AKR1C3 promotes radioresistance in prostate cancer cells. **A** Colony formation assay in LNCaP and LNCaP-AKR1C3 cell lines treated with 2 Gy of IR. **B** Colony formation assays in 22Rv1 and 22Rv1-siAKR1C3 cell lines treated with 2 Gy of IR. **C** Comet assays in LNCaP and LNCaP-AKR1C3 cell lines treated with 5 Gy of IR. **D** Comet assays in 22Rv1 and 22Rv1-siAKR1C3 cell lines treated with 5 Gy of IR. **E** Western blot analysis of γ-H2AX expression in 22Rv1 and 22Rv1-siAKR1C3 cell lines at five different time points following exposure to 5 Gy of IR. **F** Western blot analysis of AKR1C3 expression in PC-3 cells following RT. **G** Colony formation assays in PC-3 and PC-3-AKR1C3 cell lines treated with 2 Gy of IR. **H** Western blot analysis of γ-H2AX expression in PC-3 and PC-3-siAKR1C3 cell lines at five different time points following exposure to 5Gy of IR. Statistical significance: *p < 0.05, **p < 0.01, ***p < 0.001, ns not significant.

To explore the relationship between AKR1C3 and radioresistance, we chose three PCa cell lines with different AKR1C3 and AR expression levels: LNCaP (low AKR1C3 expression and positive AR expression), 22Rv1 (high AKR1C3 expression and positive AR expression) and PC-3 (normal AKR1C3 expression and negative AR expression). We overexpressed AKR1C3 in LNCaP cells and knocked it down in 22Rv1 cells. Under the same radiation dose, the LNCaP-AKR1C3 and 22Rv1-siNC groups presented greater colony numbers than did the LNCaP-pCMV6 and 22Rv1-siAKR1C3 groups, respectively (Fig. 2A, B). Additionally, the comet tail length, an indicator of DNA damage, was shorter in the LNCaP-AKR1C3 and 22Rv1-siNC groups than in the LNCaP-pCMV6 and 22Rv1-siAKR1C3 groups (Fig. 2C, D). Furthermore, at time points ranging from 0.5 to 8 h after IR treatment, the expression of γ-H2AX, a marker of DNA damage, was lower in the 22Rv1 group than in the 22Rv1-siAKR1C3 group (Fig. 2E). Given that AKR1C3 can generate androgens to activate AR, we next sought to investigate whether AKR1C3 could also mediate radioresistance in AR-negative prostate cancer cell lines, which lack androgen receptor signalling. In PC-3 cells, AKR1C3 expression was upregulated following radiation exposure (Fig. 2F) and under the same radiation dose, AKR1C3 overexpression resulted in a higher colony formation rate (Fig. 2G) and reduced γ-H2AX expression at the same time points (Fig. 2H). These findings indicate that AKR1C3 contributes to radioresistance in both AR-positive and AR-negative prostate cancer cells.

### AKR1C3 drives radioresistance by upregulating the DDR protein UBE2T

**Fig. 3:**
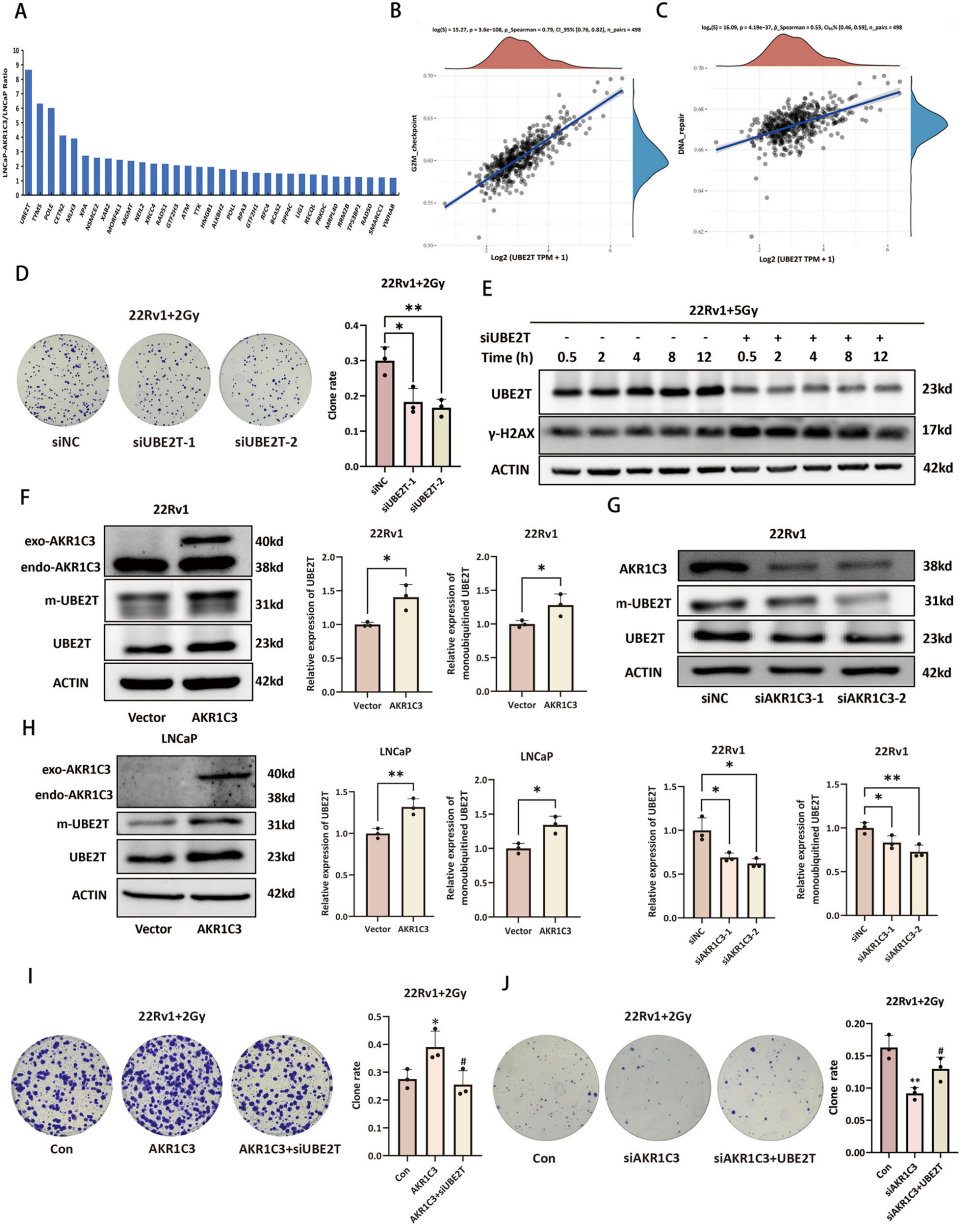
AKR1C3 drives radioresistance by upregulating the DDR protein UBE2T. **A** A bar chart of the proteomic data reveals upregulated DDR proteins in the LNCaP-AKR1C3 cell line relative to the parental controls. **B** A scatter plot showing the correlation between UBE2T expression and the expression of G2/M checkpoint genes. **C** A scatter plot showing the correlation between UBE2T expression and the expression of DNA repair genes. **D** Colony formation assays of 22Rv1 and 22Rv1-siUBE2T cell lines treated with 2Gy of IR. **E** Western blot analysis of γ-H2AX expression in 22Rv1 and 22Rv1-siUBE2T cell lines at five different time points following exposure to 5 Gy of IR. **F**–**H** Western blot analysis of UBE2T and m-UBE2T expression in the 22Rv1, 22Rv1-siAKR1C3, 22Rv1-AKR1C3, LNCaP and LNCaP-AKR1C3 cell lines. **I** Colony formation assays in 22Rv1, 22Rv1-AKR1C3 and 22Rv1-AKR1C3-siUBE2T cell lines treated with 2Gy of IR. **J** Colony formation assays in 22Rv1, 22Rv1-siAKR1C3 and 22Rv1-siAKR1C3-UBE2T cell lines treated with 2 Gy of IR. Statistical significance: *p < 0.05, **p < 0.01, ***p < 0.001 vs. the Con group; #*p* < 0.05, ##*p* < 0.01, ###*p* < 0.001 vs. the AKR1C3 group; ns not significant.

To investigate the molecular mechanism underlying AKR1C3-induced radioresistance, we conducted proteomic analysis of LNCaP and LNCaP-AKR1C3 cell lines. By comparing the upregulated DEGs with a set of DDR proteins, we found that AKR1C3 overexpression upregulated numerous DDR proteins, with UBE2T showing the most pronounced upregulation (Fig. 3A). UBE2T, a core factor in DNA interstrand crosslink repair and its monoubiquitinated form, monoubiquitinated UBE2T (m-UBE2T), which regulates its stability and function, have been shown to play a key role in radioresistance in liver cancer and non-small cell lung cancer [7, 30]. TCGA RNA-seq data from prostate cancer patients revealed a positive correlation between the expression of UBE2T and genes involved in the G2/M checkpoint and DNA repair pathways (Fig. 3B, C). Furthermore, knockdown of UBE2T in the 22Rv1 cell line significantly reduced colony formation (Fig. 3D) and increased γ-H2AX expression at the same time points following radiation (Fig. 3E). Western blot analysis revealed that UBE2T and m-UBE2T expression increased or decreased in parallel with AKR1C3 expression, indicating that UBE2T is a downstream DDR protein regulated by AKR1C3 (Fig. 3F–H). Additionally, colony formation assays demonstrated that the overexpression of AKR1C3 in 22Rv1 cells enhanced their resistance to radiotherapy, whereas the knockdown of UBE2T abolished AKR1C3-mediated radioresistance (Fig. 3I). Finally, rescue experiments showed that reintroduction of UBE2T enhanced radioresistance in prostate cancer cells (Fig. 3J). These findings indicate that AKR1C3 can promote radioresistance by upregulating UBE2T.

### AKR1C3 enhances UBE2T transcriptional activation by promoting Nrf2 nuclear translocation

**Fig. 4:**
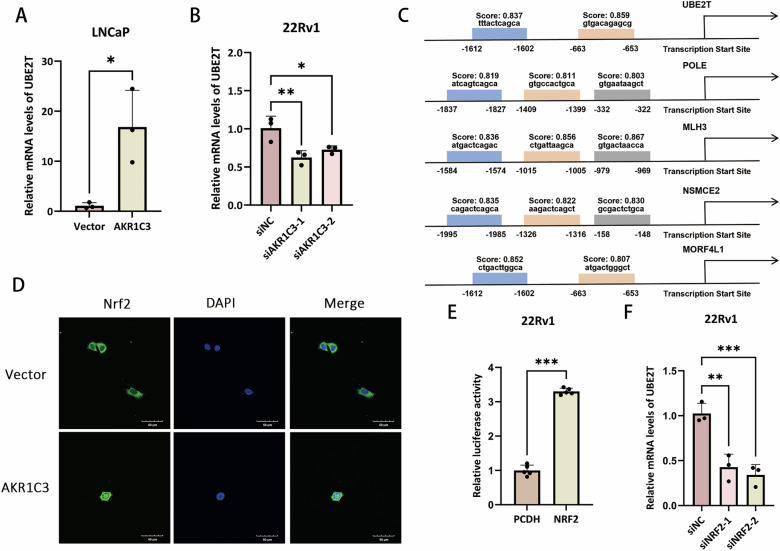
AKR1C3 enhances UBE2T transcriptional activation by promoting Nrf2 nuclear translocation. **A**, **B** RT-qPCR results of relative UBE2T mRNA levels in LNCaP, LNCaP-AKR1C3, 22Rv1 and 22Rv1-siAKR1C3 cell lines. **C** JASPAR prediction of Nrf2 binding to the top five upregulated DDR promoter regions from proteomics. **D** Immunofluorescence analysis of Nrf2 nuclear translocation in 22Rv1 and 22Rv1-AKR1C3 cell lines. **E** Dual-luciferase reporter assay to evaluate Nrf2 transcriptional activity on UBE2T. **F** RT-qPCR results of relative UBE2T mRNA levels in 22Rv1, 22Rv1-siNRF2-1 and 22Rv1-siNRF2-2. Statistical significance: *p < 0.05, **p < 0.01, ***p < 0.001 vs. the Con group.

To investigate the specific mechanism by which AKR1C3 upregulates UBE2T expression, RT-qPCR results indicate that AKR1C3 enhances UBE2T expression through transcriptional regulation, possibly by activating specific transcription factors involved in UBE2T promoter activity (Fig. 4A, B). Additionally, motif scanning in JASPAR of the top five DDR genes upregulated by AKR1C3 revealed antioxidant response element sites for Nrf2 in each promoter (Fig. 4C). Immunofluorescence results showed that overexpression of AKR1C3 in 22Rv1 cells promotes the nuclear translocation of Nrf2 (Fig. 4D). Consistently, luciferase assays showed that Nrf2 binds to the UBE2T promoter region (Fig. 4E). Meanwhile, RT-qPCR results indicated that knockdown of NRF2 led to a significant reduction in UBE2T mRNA levels. These results suggest that AKR1C3 enhances UBE2T transcriptional activation by promoting Nrf2 nuclear translocation.

### Proteomics reveals that AKR1C3 upregulates oxphos-related proteins in prostate cancer

**Fig. 5:**
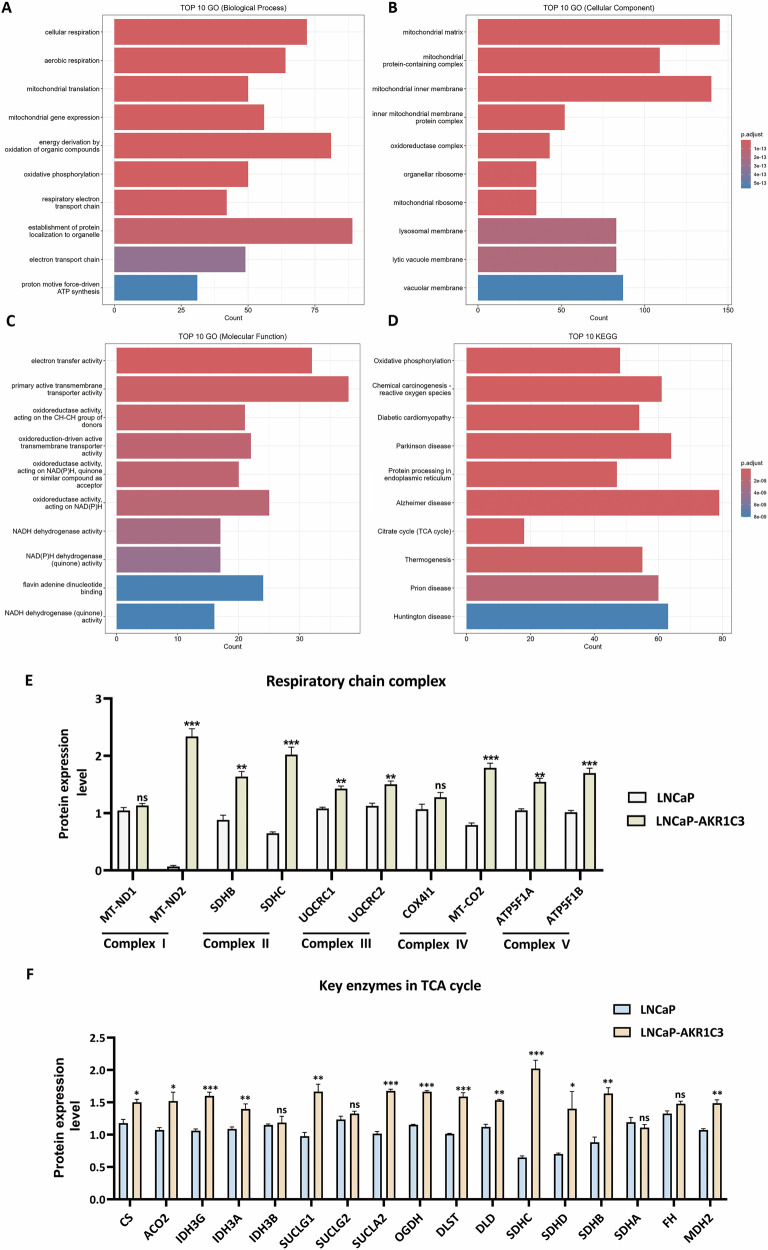
Proteomic analysis reveals that AKR1C3 upregulates OXPHOS-related proteins in prostate cancer. **A**–**D** GO and KEGG enrichment analyses of upregulated genes in LNCaP-AKR1C3 cells compared with control cells. **E** Relative expression levels of key respiratory chain complex proteins in LNCaP and LNCaP-AKR1C3 cells. **F** Relative expression levels of key TCA cycle enzymes in LNCaP and LNCaP-AKR1C3 cells. Statistical significance: *p < 0.05, **p < 0.01, ***p < 0.001 vs. the Con group.

To elucidate the pathway through which AKR1C3 affects the expression of DDR proteins, we conducted an enrichment analysis of our proteomic data. Notably, the results demonstrated that OXPHOS-related pathways were significantly enriched in the proteins upregulated following AKR1C3 overexpression, consistently ranking at the top (Fig. 5A–D). Further analysis revealed that many key enzymes in the tricarboxylic acid (TCA) cycle and many key components in the respiratory chain complex were enriched in the proteins in the LNCaP-AKR1C3 cells compared to the control cells (Fig. 5E, F), indicating that AKR1C3 plays a critical role in regulating OXPHOS-related proteins.

### AKR1C3 promotes oxidative phosphorylation and OXPHOS inhibition abrogates AKR1C3-mediated upregulation of UBE2T

**Fig. 6:**
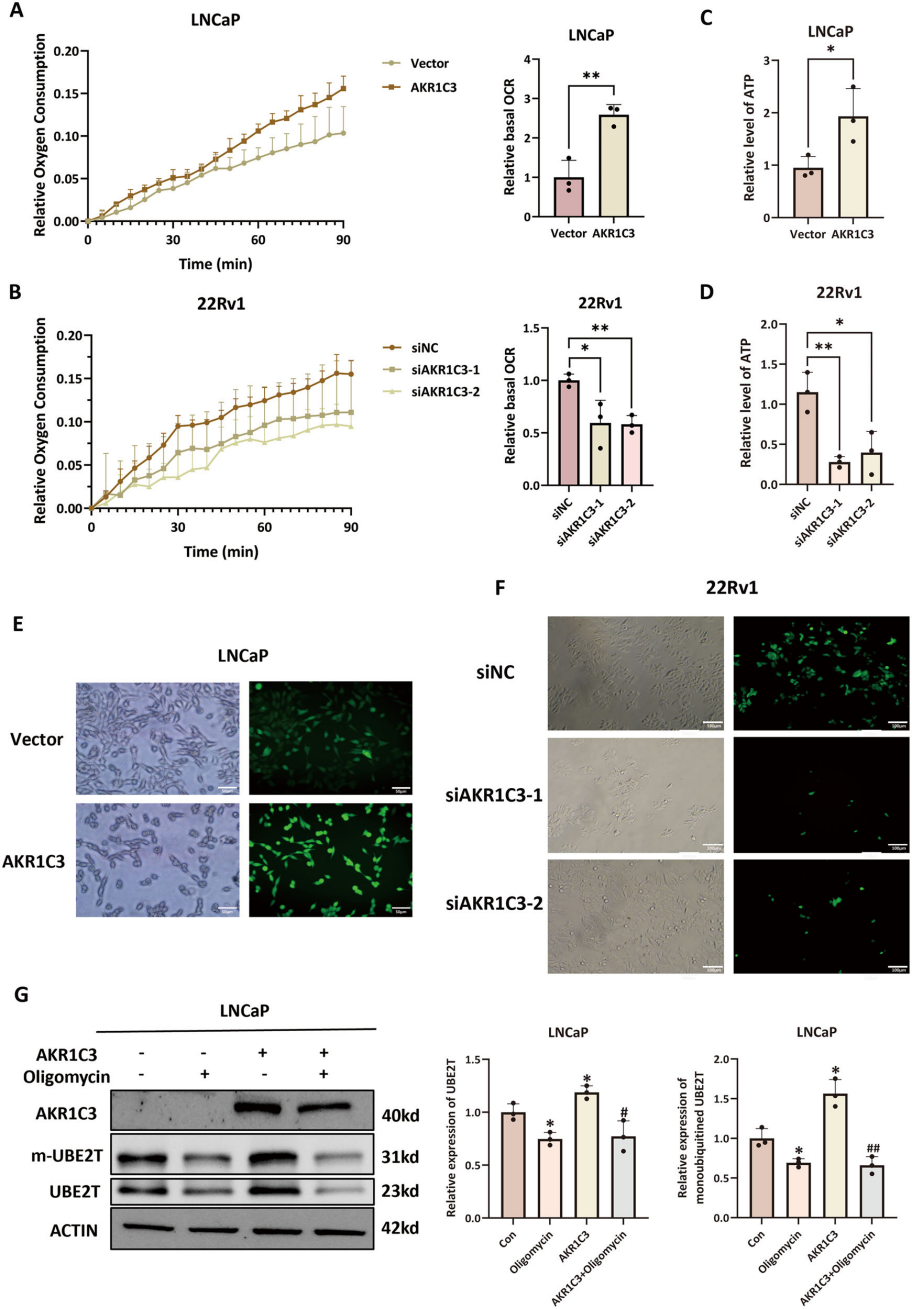
AKR1C3 promotes oxidative phosphorylation and OXPHOS inhibition abrogates AKR1C3-mediated upregulation of UBE2T. **A**, **B** Line chart showing the basal OCR of the 22Rv1, 22Rv1-siAKR1C3, LNCaP and LNCaP-AKR1C3 cell lines. **C**, **D** Bar chart showing the relative ATP content in the 22Rv1, 22Rv1-siAKR1C3, LNCaP and LNCaP-AKR1C3 cell lines. **E**, **F** ROS levels in 22Rv1, 22Rv1-siAKR1C3, LNCaP and LNCaP-AKR1C3 cell lines. **G** Western blot analysis of UBE2T expression in LNCaP and LNCaP-AKR1C3 cells with or without 1 μM oligomycin treatment. Statistical significance: *p < 0.05, **p < 0.01, ***p < 0.001 vs. the Con group; #*p* < 0.05, ##*p* < 0.01, ###*p* < 0.001 vs. the AKR1C3 group; ns = not significant.

To test whether AKR1C3 enhances OXPHOS in prostate cancer cells, we measured basal OCR, cellular ATP content and ROS levels in LNCaP, LNCaP-AKR1C3, 22Rv1 and 22Rv1-siAKR1C3 cell lines. Results showed that AKR1C3 overexpression increased OCR, ATP content and ROS levels, whereas AKR1C3 knockdown produced the opposite changes (Fig. 6A–F), confirming that AKR1C3 promotes OXPHOS in prostate cancer cells. Furthermore, we employed 1 μM oligomycin, an OXPHOS inhibitor, to treat LNCaP and LNCaP-AKR1C3 cells and performed WB analysis. The results indicated that AKR1C3 increased UBE2T expression; however, this regulatory effect was abolished upon OXPHOS inhibition (Fig. 6G). Collectively, these data indicate that AKR1C3 drives mitochondrial metabolic reprogramming characterised by increased oxidative phosphorylation, leading to increased ATP and ROS production. This OXPHOS-dependent metabolic state is required for AKR1C3-mediated upregulation of the DDR protein UBE2T in prostate cancer.

### AKR1C3 promotes oxidative phosphorylation in prostate cancer cells by binding to PKM2 and accelerating its degradation

**Fig. 7:**
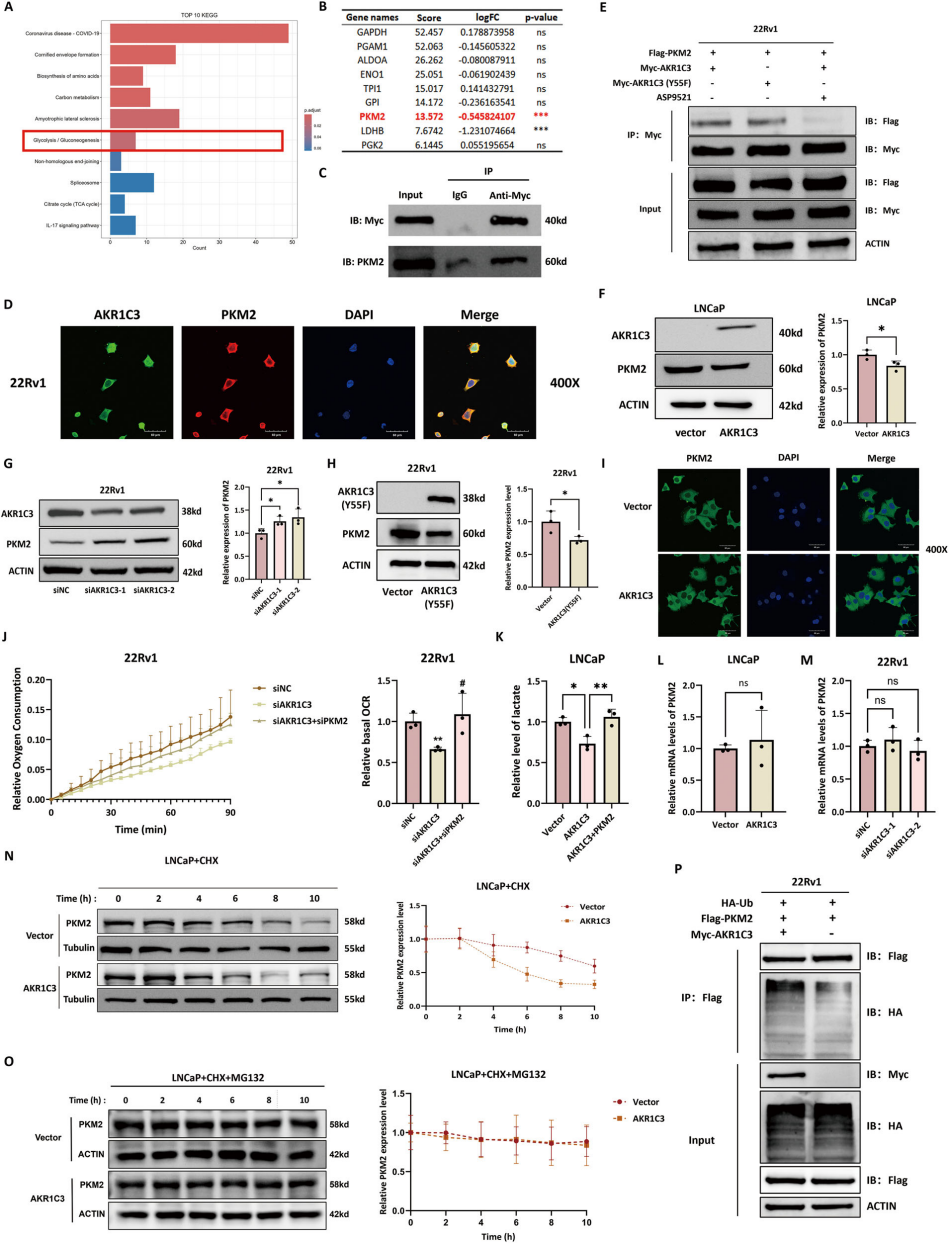
AKR1C3 promotes oxidative phosphorylation in prostate cancer cells by binding to PKM2 and accelerating its degradation. **A** Bar chart showing the KEGG enrichment results for AKR1C3-interacting proteins. **B** Table presenting the binding scores from CoIP-mass spectrometry analysis and logFC values from proteomic analysis of glycolysis-related proteins in the LNCaP-AKR1C3 cell line. **C** CoIP assay results presenting the interactions between AKR1C3 and PKM2 in LNCaP-AKR1C3 cells. **D** Immunofluorescence images showing the colocalization of AKR1C3 and PKM2 in 22Rv1 cells. **E** CoIP analysis of the interaction between AKR1C3, AKR1C3 (Y55F), AKR1C3 + ASP9521 treatment and PKM2 in 22Rv1 cells. **F**, **G** Western blot analysis of PKM2 expression in LNCaP, LNCaP-AKR1C3, 22Rv1 and 22Rv1-siAKR1C3 cell lines. **H** Western blot analysis of PKM2 expression in 22Rv1, 22Rv1-AKR1C3 (Y55F) cell lines. **I** Immunofluorescence analysis of PKM2 nuclear translocation in 22Rv1 and 22Rv1-AKR1C3 cell lines. **J** Line chart showing the basal OCR of the 22Rv1, 22Rv1-siAKR1C3 and 22Rv1-siAKR1C3+siPKM2 cell lines. **K** Lactate production in LNCaP, LNCaP-AKR1C3 and LNCaP-AKR1C3-PKM2 cell lines. **L**, **M** RT-qPCR results of relative PKM2 mRNA levels in LNCaP, LNCaP-AKR1C3, 22Rv1 and 22Rv1-siAKR1C3 cell lines. **N** CHX assays showing the PKM2 degradation dynamics in LNCaP and LNCaP-AKR1C3 cell lines. **O** MG132 rescue experiment showing the PKM2 degradation dynamics in LNCaP and LNCaP-AKR1C3 cell lines. **P** CoIP analysis of the interaction between AKR1C3, Flag-tagged PKM2 and HA-tagged ubiquitin (HA-Ub) in 22Rv1 and 22Rv1-AKR1C3 cell lines. Statistical significance: *p < 0.05, **p < 0.01, ***p < 0.001, ns not significant vs. the Con group; #*p* < 0.05, ##*p* < 0.01, ###*p* < 0.001 vs. the siAKR1C3 group; ns not significant.

To further explore the mechanism by which AKR1C3 regulates OXPHOS in prostate cancer, we overexpressed AKR1C3 in LNCaP cells and performed CoIP followed by mass spectrometry to identify AKR1C3-interacting proteins. KEGG enrichment analysis of the precipitated proteins revealed significant enrichment of the glycolysis pathway (Fig. 7A). Notably, PKM2, a key glycolytic enzyme, was identified as a potential binding partner of AKR1C3 and its protein expression level was decreased in the LNCaP-AKR1C3 cell line (Fig. 7B). Consistent with these findings, our CoIP results demonstrated an interaction between AKR1C3 and PKM2 in LNCaP cells and we observed colocalization of AKR1C3 and PKM2 in 22Rv1 cells (Fig. 7C, D).

To further investigate the binding region of AKR1C3 with PKM2 and whether this interaction depends on AKR1C3’s enzymatic activity, we utilised AKR1C3 enzymatic mutants and selective inhibitors. The Y55F mutation is known to significantly impair or nearly abolish AKR1C3’s enzymatic activity [31]. was introduced, while ASP9521, a competitive inhibitor of AKR1C3, is expected to target its substrate-binding pocket [32, 33]. The results revealed that the AKR1C3(Y55F) mutant also could bind to PKM2, whereas treatment with ASP9521 reduced the interaction between AKR1C3 and PKM2 (Fig. 7E). These findings indicate that the AKR1C3–PKM2 interaction does not rely on AKR1C3’s catalytic activity and that the binding site may be located within the substrate-binding region.

Additionally, Western blot analysis further confirmed that AKR1C3 overexpression significantly reduced PKM2 protein levels, while AKR1C3 knockdown led to an increase in PKM2 expression (Fig. 7F, G). Interestingly, overexpression of the AKR1C3 (Y55F) mutant also significantly decreased PKM2 protein levels (Fig. 7H). These findings further demonstrate that AKR1C3 regulates PKM2 expression independently of its enzymatic activity.

Given that the oligomeric state of PKM2 is closely linked to its activity, the dimer exhibits low enzymatic activity and functions in the nucleus for transcriptional regulation, whereas the tetramer is predominantly localised in the cytoplasm and exhibits high enzymatic activity. We further investigated the specific oligomeric state of PKM2 during its interaction with AKR1C3. Immunofluorescence results showed that AKR1C3 expression did not affect the nuclear translocation of PKM2 (Fig. 7I). Additionally, AlphaFold3 analysis revealed that AKR1C3 exhibited the highest protein-protein interaction scores with the PKM2 tetramer (iPTM=0.64, PTM = 0.7), suggesting that AKR1C3 is more likely to interact with the PKM2 tetramer (Fig. S1A–F). Based on this, further analysis of the binding site revealed that lysine 33 (Lys33), glutamic acid 127 (Glu127) and asparagine 134 (Asn134) on the surface of AKR1C3 are potential interaction sites with PKM2 (Fig. S1G).

Functionally, PKM2 knockdown rescued the basal OCR reduction caused by AKR1C3 depletion and PKM2 overexpression rescued the decrease in lactate levels induced by AKR1C3 overexpression (Fig. 7J, K). Mechanistically, RT-qPCR revealed no statistically significant change in PKM2 mRNA (Fig. 7L, M), while CHX chase assays indicated accelerated PKM2 degradation upon AKR1C3 overexpression in LNCaP cells (Fig. 7N), suggesting that AKR1C3 suppresses PKM2 by accelerating its degradation rather than inhibiting its transcription. Further MG132 rescue experiments and CoIP assays revealed that AKR1C3 promotes PKM2 degradation through the ubiquitin-proteasome pathway (Fig. 7O, P). These results indicate that AKR1C3 promotes OXPHOS in prostate cancer by binding to and accelerating PKM2 degradation mainly via the ubiquitin-proteasome pathway.

### In vivo evaluation of AKR1C3’s role in radiotherapy resistance

**Fig. 8:**
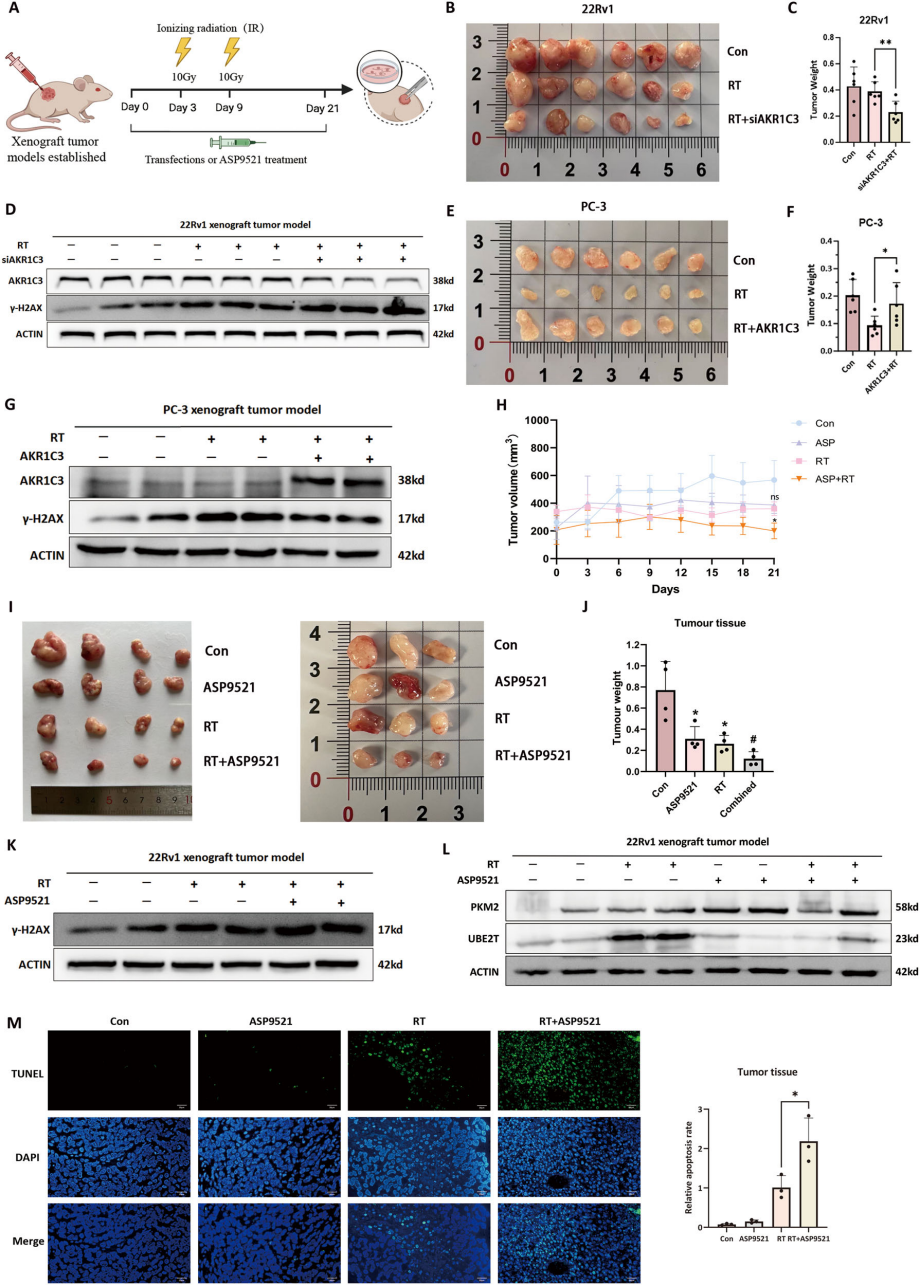
In vivo evaluation of AKR1C3’s role in radiotherapy resistance. **A** Experimental schematic of xenograft tumour models establishment and treatments. **B** Representative images of tumours from the first batch of 22Rv1 xenograft tumour model for each treatment group at day 21. **C** Tumour weight quantification for each treatment group from the first batch of 22Rv1 xenograft tumour model. **D** Western blot analysis of AKR1C3 and γ-H2AX expression levels in the tumour tissue from the first batch of 22Rv1 xenograft model. **E** Representative images of tumours from the PC-3 xenograft tumour model for each treatment group at day 21. **F** Tumour weight quantification for the PC-3 xenograft tumour model. **G** Western blot analysis of AKR1C3 and γ-H2AX expression in the tumour tissue from the PC-3 xenograft model. **H** Tumour volume curves for each treatment group in the second batch of 22Rv1 xenograft tumour model. **I** Representative images of tumours from the second batch of 22Rv1 xenograft tumour model for each treatment group at day 21. **J** tumour weight quantification for each treatment group from the second batch of 22Rv1 xenograft tumour model. **K** Western blot analysis of γ-H2AX expression levels in the tumour tissue from the second batch of 22Rv1 xenograft model. **L** Western blot analysis of PKM2 and UBE2T expression levels in the tumour tissue from the second batch of 22Rv1 xenograft model. **M** TUNEL staining of tumour tissue for each treatment group from the second batch of 22Rv1 xenograft model. Statistical significance: *p < 0.05, **p < 0.01, ***p < 0.001, ns = not significant vs. the Con group; #*p* < 0.05, ##*p* < 0.01, ###*p* < 0.001 vs. the RT group; ns not significant.

To evaluate the role of AKR1C3 in radioresistance in vivo, we established 22Rv1 and PC-3 xenograft tumor models in BALB/c-nu mice. We assessed the impact of AKR1C3 on radioresistance through overexpression, knockdown and inhibitor treatment (Fig. 8A). Consistent with the in vitro results, we found that in the 22Rv1 xenograft tumor model, AKR1C3 knockdown combined with radiotherapy resulted in a better antitumor effect compared to radiotherapy alone (Fig. 8B), as evidenced by reduced tumor weight and increased γ-H2AX levels (Fig. 8C, D), indicating enhanced radiosensitivity. In the PC-3 xenograft tumor model, AKR1C3 overexpression did not significantly reduce tumor volume post radiotherapy and γ-H2AX levels were lower, indicating that AKR1C3 contributes to radioresistance (Fig. 8E–G).

We further evaluated the sensitising effect of the AKR1C3 selective inhibitor ASP9521 in combination with radiotherapy in the 22Rv1 xenograft tumor model, focusing on its ability to enhance radiosensitivity. The combined treatment group showed better antitumor effects than either treatment alone, with higher γ-H2AX levels, indicating that AKR1C3 inhibition improves prostate cancer’s response to radiotherapy (Fig. 8H–K). Moreover, we evaluated the safety of ASP9521 combined with radiotherapy and found that, compared to the radiotherapy-only group, the liver and kidney tissues showed no significant pathological changes by HE staining (Fig. S2A). Additionally, there were no significant changes observed in body weight, liver-to-body weight ratio, kidney-to-body weight ratio or spleen-to-body weight ratio (Fig. S2B–E). Western blot analysis of tumor tissues revealed that ASP9521 decreased UBE2T levels (Fig. 8L). TUNEL assay results showed that treatment with ASP9521 alone did not induce significant apoptosis in tumor tissues, whereas radiotherapy alone elicited noticeable apoptotic cell death. Combined ASP9521 and radiotherapy treatment significantly increased the number of TUNEL-positive cells compared to radiotherapy alone (Fig. 8M). These findings suggest that AKR1C3 inhibition does not directly induce tumor cell apoptosis but significantly enhances apoptosis induced by radiotherapy, thereby sensitising tumors to radiotherapy by enhancing their apoptotic response to treatment.

## Discussion

Prostate cancer radioresistance, with enhanced DNA repair capacity and cell cycle regulation, involves multiple mechanisms. So far, the pivotal central role of the AR signalling pathway is widely recognised in prostate cancer [34]. Upon dimerisation and nuclear translocation, AR activation leads to the upregulation of DDR genes such as RAD51 and KU70, activating multiple DNA repair pathways [11]. Androgens, the key ligand of AR activation, are biosynthesized through multiple pathways: the classical pathway involves de novo synthesis from cholesterol; the alternative pathway converts adrenal-derived weak androgens such as androstenedione and dehydroepiandrosterone into potent androgens such as testosterone and dihydrotestosterone; and the backdoor pathway produces dihydrotestosterone directly from progesterone metabolites without testosterone as an intermediate. In all these pathways, AKR1C3 acts as a critical downstream enzyme in the synthesis of intratumoral testosterone or dihydrotestosterone [35]. Consequently, AKR1C3 serves as a key enzyme in endogenous androgen synthesis and catalyses the production of androgens in prostate cancer, thereby sustaining AR pathway activation and indirectly contributing to radioresistance via its enzymatic activity [36]. However, AKR1C3 also promotes prostate cancer progression through nonenzymatic mechanisms. Studies have shown that AKR1C3 can bind to Siah2 and AR-V7 and inhibit their ubiquitination and degradation [12, 13]. In this study, we identify AKR1C3 as a central driver of prostate cancer radioresistance by binding to and accelerating PKM2 degradation, thereby enhancing OXPHOS and upregulating the DDR protein UBE2T. Additionally, we note that the non-enzymatic function of AKR1C3 promotes radioresistance in both AR-positive and AR-negative prostate cancer cells. These effects are validated in in vivo xenograft models, positioning AKR1C3 as a promising therapeutic target.

Cancer cells typically rely on aerobic glycolysis to meet their rapid energy demands [37]. Pyruvate kinase (PK) is a key rate-limiting enzyme in the glycolytic pathway, responsible for converting phosphoenolpyruvate to pyruvate, thereby regulating glycolytic flux [38]. In mammals, there are four isoforms of pyruvate kinase, with PKM2 showing significantly higher expression in most tumors [39]. Moreover, different oligomeric states of PKM2 play crucial roles in metabolic regulation. The dimeric form of PKM2 has lower enzymatic activity and can translocate to the nucleus, where it participates in transcriptional regulation [40, 41] while the tetrameric form of PKM2 has higher enzymatic activity, predominantly residing in the cytoplasm and driving glycolysis [42]. In our experiments, we note that AKR1C3 interacts with PKM2 and promotes its ubiquitin-dependent degradation. More importantly, we observed that AKR1C3 binds more stably to the PKM2 tetramer and AKR1C3 overexpression does not affect PKM2 nuclear translocation, further indicating that AKR1C3 is more likely to interact with the PKM2 tetramer rather than its dimeric form. Due to the reduced levels of PKM2 tetramer with high glycolytic enzymatic activity, glycolytic flux in prostate cancer is decreased and metabolism is redirected toward OXPHOS. This AKR1C3–PKM2 axis-mediated metabolic switch provides a metabolic basis for radioresistance in prostate cancer. This metabolic reprogramming may enhance radioresistance through two mechanisms [1] abundant ATP produced by OXPHOS supports DNA repair and [2] moderately elevated mitochondrial ROS levels induce mild oxidative stress, adaptively increasing the DNA damage repair ability of the cancer cells.

Specifically, OXPHOS exerts its role in promoting DDR through its products, ATP and ROS. Firstly, ATP is an essential energy source for DNA repair processes and is utilised by various DNA repair enzymes [43]. OXPHOS efficiently generates large amounts of ATP, providing the necessary energy for these repair processes. Additionally, ATP supports the activation of repair proteins such as Ataxia-Telangiectasia Mutated (ATM) and Ataxia-Telangiectasia and Rad3-related (ATR) kinases and their downstream phosphorylation cascades [44]. ATP also facilitates the activity of key repair enzymes, including DNA polymerases and ligases, which are crucial for repairing single- and double-strand breaks [45]. ROS, as a byproduct of OXPHOS, acts as a signalling molecule at moderate levels in the mitochondria, participating in cellular stress responses and DNA damage response signalling [46]. Low levels of ROS can activate DDR signals through oxidative modifications, enhancing the cell’s ability to recognise and respond to DNA damage [47]. Moderately elevated ROS induce oxidative stress responses, activating antioxidant transcription factors such as NRF2, which upregulate antioxidant proteins and certain repair-associated proteins [48, 49]. Finally, studies have reported a positive correlation between UBE2T expression and oxygen levels and OXPHOS may modulate tumor oxygenation to influence UBE2T expression [50]. Consistent with our experimental results, AKR1C3 promotes OXPHOS, thereby increasing intracellular ATP and ROS levels. Additionally, AKR1C3 facilitates the nuclear translocation of NRF2, subsequently activating the transcription of UBE2T.

Currently, the inhibition of OXPHOS to increase radiotherapy sensitivity is being increasingly explored. In high-grade glioma, mitochondrial OXPHOS suppression improves the radiation response, whereas in the clinic, metformin combined with radiotherapy significantly decreases recurrence rates in prostate cancer patients [51, 52]. In this experiment, we also noted that inhibiting OXPHOS in prostate cancer cells using oligomycin abolished the UBE2T upregulation induced by AKR1C3 overexpression, thereby impairing the DNA damage repair capacity of prostate cancer cells and enhancing their sensitivity to radiotherapy.

To date, most AKR1C3 inhibitors under investigation are designed to block AKR1C3 enzymatic activity. ASP9521 is a potent, selective and orally bioavailable AKR1C3 inhibitor that competitively binds to the active site of the enzyme, making it an excellent therapeutic tool for studying AKR1C3 [33]. The results show that ASP9521 exhibits a potent radiosensitizing effect in the 22Rv1 xenograft model. More importantly, ASP9521 can disrupt the interaction between AKR1C3 and PKM2, indicating that ASP9521 not only inhibits AKR1C3 enzymatic activity by binding to its active site but also prevents the binding of proteins that interact with AKR1C3’s active site. Therefore, the development of competitive inhibitors targeting the active site of AKR1C3 may hold great promise for future therapeutic strategies.

Although our study provides preliminary evidence for the role of AKR1C3 in promoting radioresistance in prostate cancer, several limitations warrant further discussion. Firstly, we did not systematically explore the expression dynamics of AKR1C3 after radiotherapy or its potential contribution to adaptive resistance, which limits our understanding of its function throughout the radiotherapy process. Additionally, there is a gap between in vitro models and the in vivo tumour microenvironment. The in vitro experimental conditions cannot fully recapitulate the complex factors of prostate cancer in vivo, such as hypoxia, immune cell infiltration and tumour-stroma interactions, all of which may significantly influence the function of AKR1C3 and its role in mediating radioresistance. Given these unaccounted factors, the clinical relevance and generalisability of our experimental findings remain limited.

In summary, by integrating clinical data from the TCGA and GEO databases and validating findings through in vitro and in vivo experiments, this study systematically elucidates the critical role of the nonenzymatic functions of AKR1C3 in prostate cancer radioresistance, providing a new target for overcoming radioresistance in prostate cancer. Specifically, AKR1C3 can bind to PKM2 and accelerate its degradation, thereby inhibiting glycolytic flux and enhancing OXPHOS. The increase in OXPHOS boosts ATP and ROS production. ATP provides the necessary energy for DNA damage repair, while ROS further promotes NRF2 nuclear translocation, activating the transcription of UBE2T, thereby driving radioresistance in prostate cancer.

## Supplementary information

Western blot raw data

Proteomics raw data

Supplementary Tables

Figure S1

Figure S2

Supplementary Figures Caption

## Data Availability

Publicly available datasets were analysed in this study. These data can be found in the TCGA and GEO databases. The proteomics data generated in this study are provided in the supplementary files (10.6084/m9.figshare.31189081).
