# Supplementary material for: AKR1C3–PKM2–oxidative phosphorylation axis drives prostate cancer radioresistance via UBE2T upregulation
Source: Cell Death Dis. 2026 Mar 30;17(1):433. doi: 10.1038/s41419-026-08666-5 (PMC13158291; doi:10.1038/s41419-026-08666-5)
Supplement: Supplementary file 1 — Western blot raw data [file 41419_2026_8666_MOESM1_ESM.pdf]

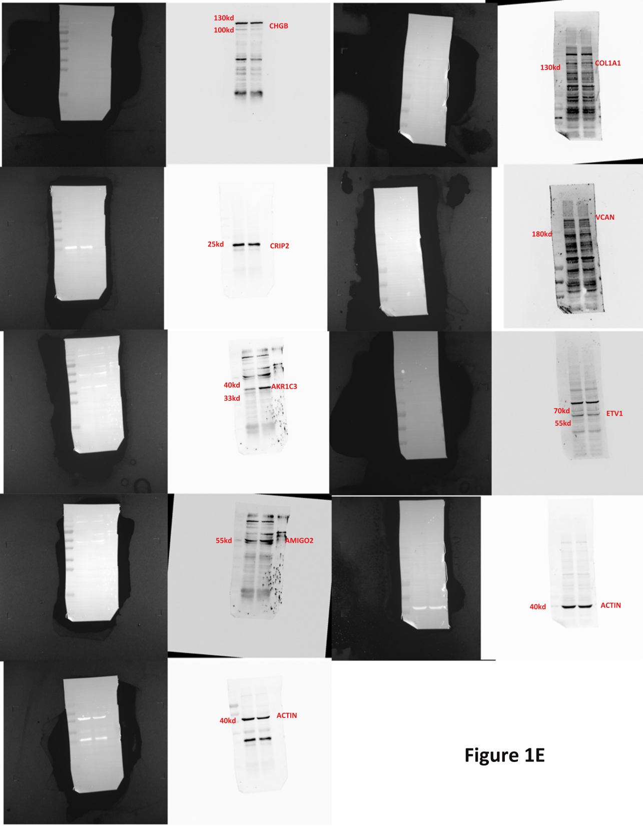
130kd
100kd
CHGB
130kd
COL1A1
25kd
CRIP2
VCAN
180kd
40kd
33kd
AKR1C3
70kd
55kd
ETV1
55kd
AMIGO2
40kd
ACTIN
40kd
ACTIN

Figure 1E

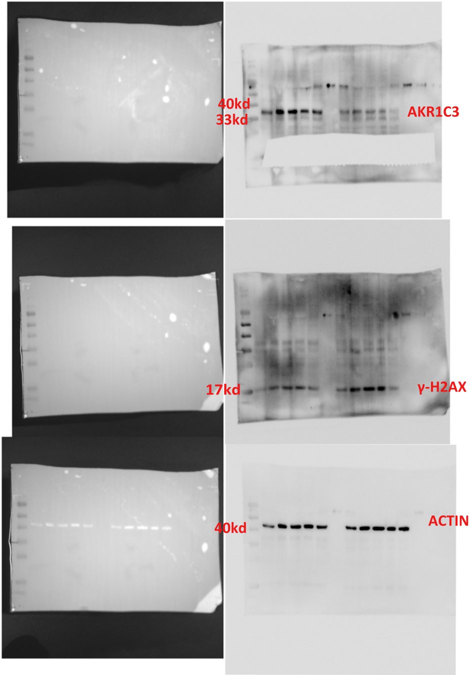

Figure 2E

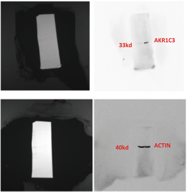

Figure 2F

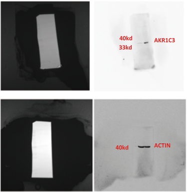

Figure 2F

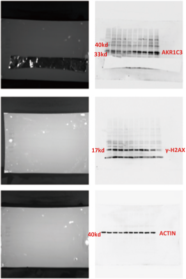

Figure 2H

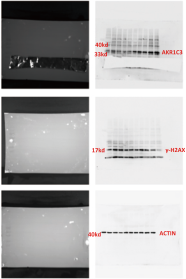

Figure 2H

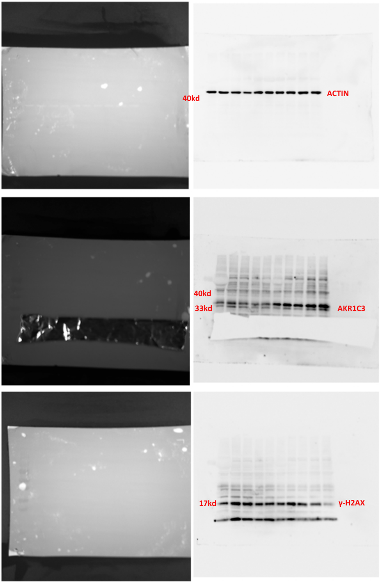

Figure 3E

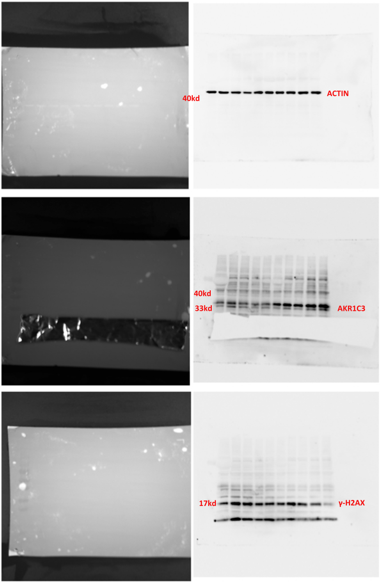

Figure 3E

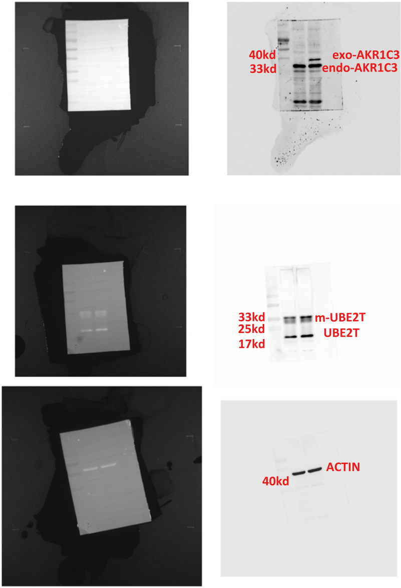
40kd
33kd
exo-AKR1C3
endo-AKR1C3
33kd
25kd
17kd
m-UBE2T
UBE2T
ACTIN
40kd

Figure 3F

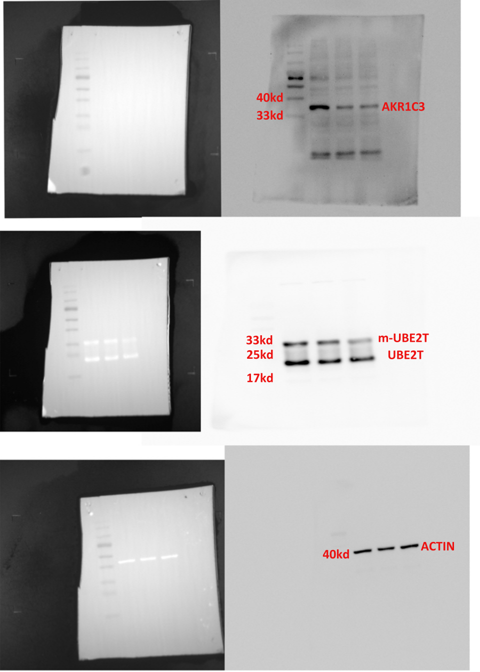

Figure 3G

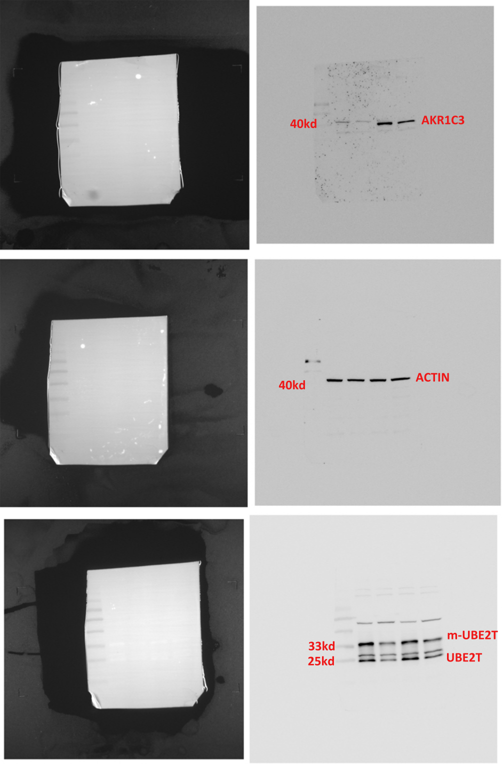

Figure 6G

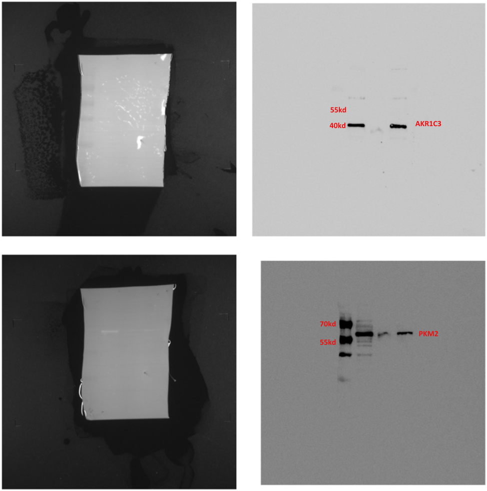

Figure 7C

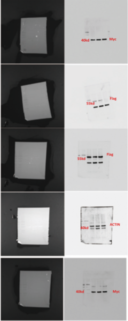

Figure 7E

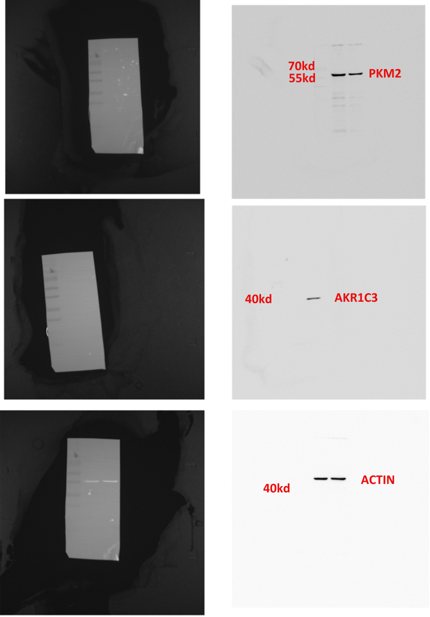

Figure 7F

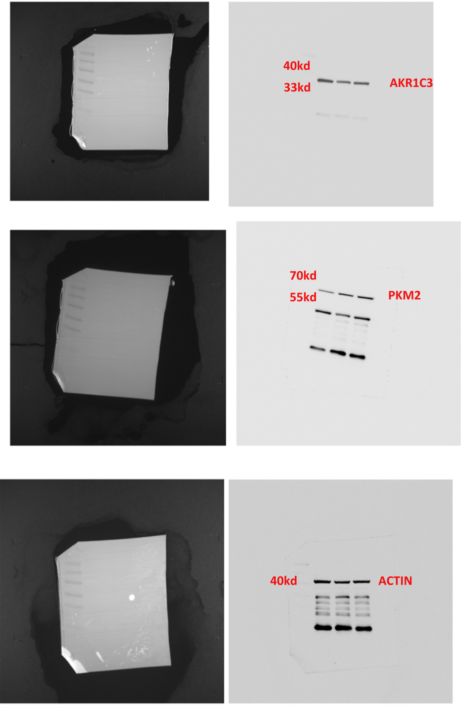

Figure 7G

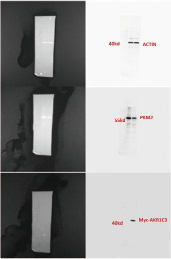

Figure 7H

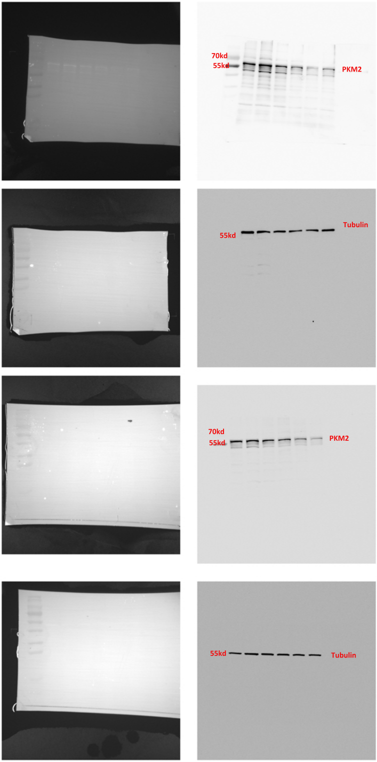

Figure 7N

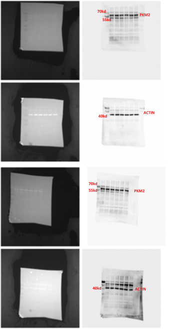

Figure 7O

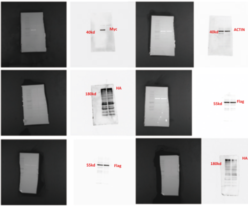

Figure 7P

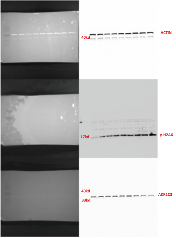

Figure 8D

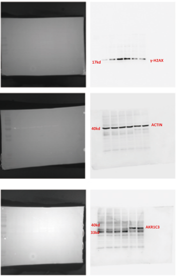

Figure 8G

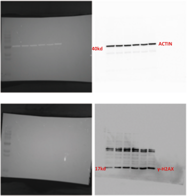

Figure 8K

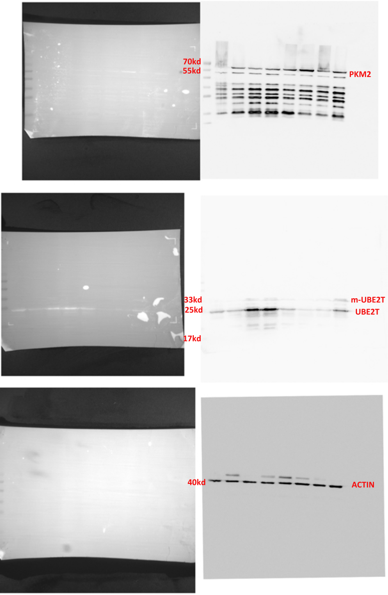

Figure 8L
