# Supplementary figures and images for: AKR1C3–PKM2–oxidative phosphorylation axis drives prostate cancer radioresistance via UBE2T upregulation
Source: Cell Death Dis. 2026 Mar 30;17(1):433. doi: 10.1038/s41419-026-08666-5 (PMC13158291; doi:10.1038/s41419-026-08666-5)

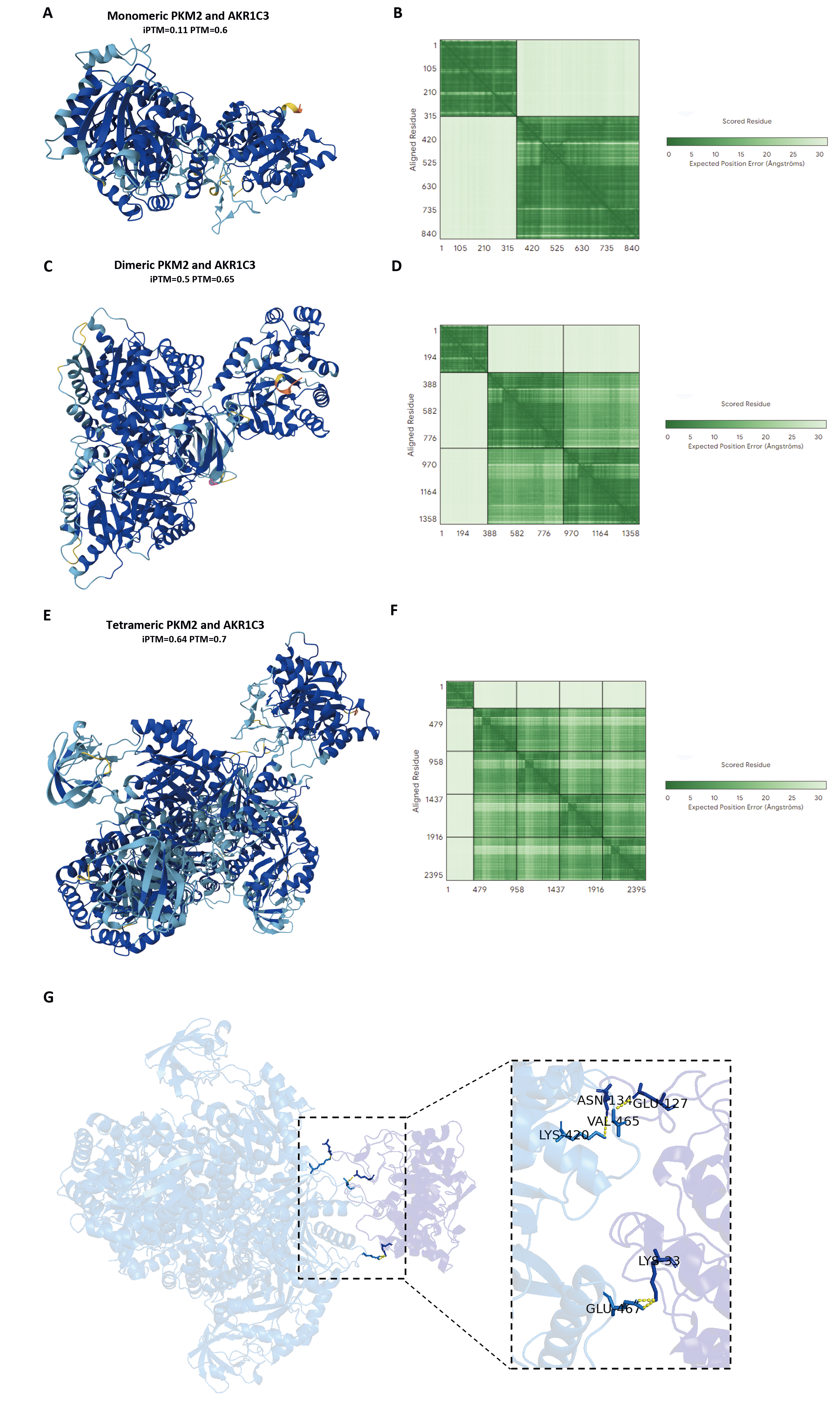

Supplement: Supplementary file 4 — Figure S1 [file 41419_2026_8666_MOESM4_ESM.png]

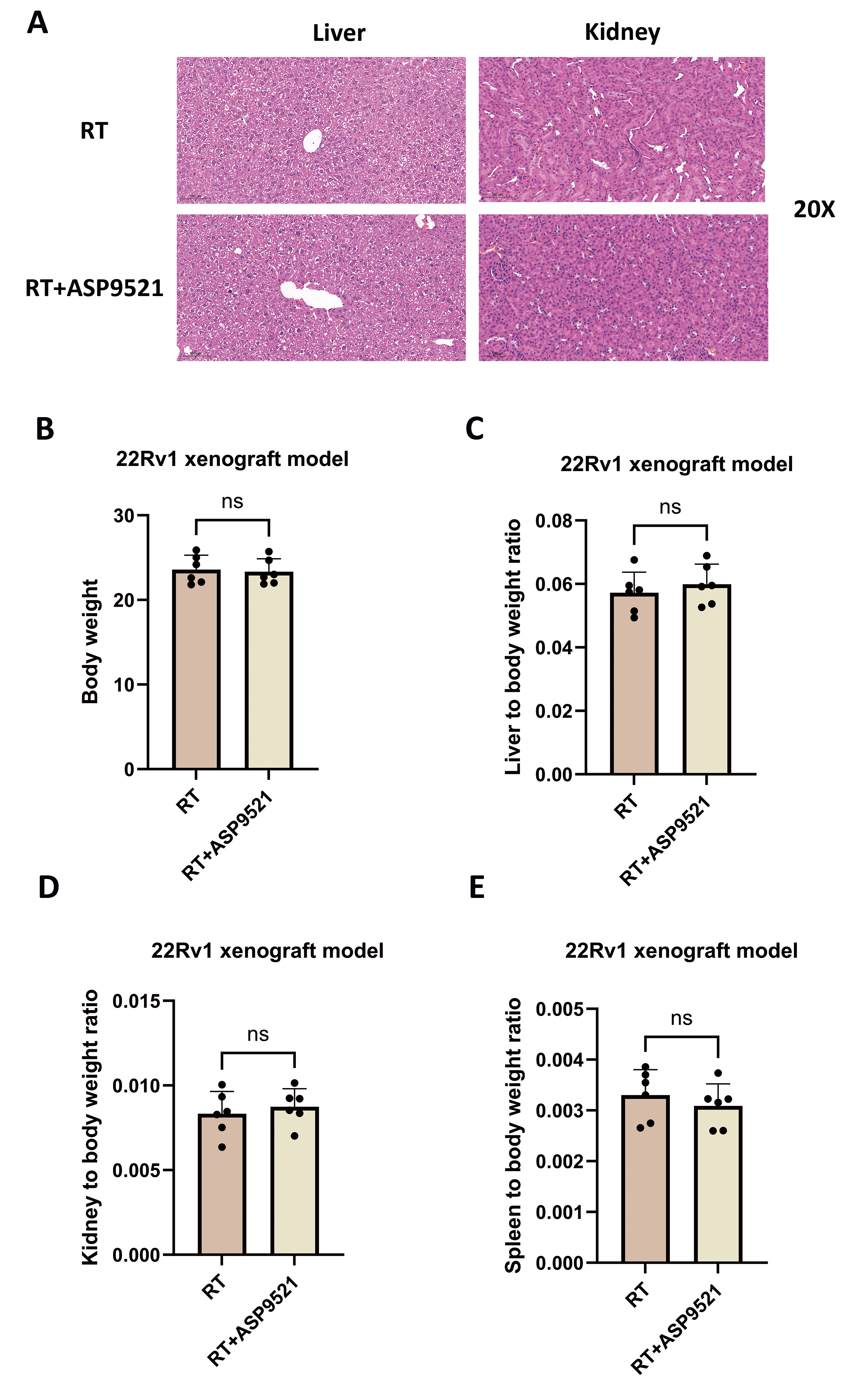

Supplement: Supplementary file 5 — Figure S2 [file 41419_2026_8666_MOESM5_ESM.png]
