# Supplementary material for: AKR1C3–PKM2–oxidative phosphorylation axis drives prostate cancer radioresistance via UBE2T upregulation
Source: Cell Death Dis. 2026 Mar 30;17(1):433. doi: 10.1038/s41419-026-08666-5 (PMC13158291; doi:10.1038/s41419-026-08666-5)
Supplement: Supplementary file 6 — Supplementary Figures Caption [file 41419_2026_8666_MOESM6_ESM.docx]

**Figure S1. AKR1C3 and PKM2 protein-protein interaction prediction.** (A-B) Monomeric PKM2 and AKR1C3 interaction prediction. (C-D) Dimeric PKM2 and AKR1C3 interaction prediction. (E-F) Tetrameric PKM2 and AKR1C3 interaction prediction. (G) Interaction site analysis of AKR1C3 with the PKM2 tetramer.

**Figure S2. Safety Evaluation of ASP9521 Combined with Radiotherapy.** (A) HE staining of liver and kidney from mice treated with RT alone or RT combined with ASP9521. (B) Body weight quantification of tumor-bearing mice over the course of the treatment with RT and/or ASP9521. (C) Liver-to-body weight ratio in mice treated with RT and/or ASP9521. (D) Kidney-to-body weight ratio in mice treated with RT and/or ASP9521. (E) Spleen-to-body weight ratio in mice treated with RT and/or ASP9521. Statistical significance: *p < 0.05, **p < 0.01, ***p < 0.001, ns = not significant vs. the RT group.
